# Palliative hysterectomy for vaginal bleeding from breast cancer metastatic to the uterus

**DOI:** 10.3332/ecancer.2018.811

**Published:** 2018-02-14

**Authors:** Amnon A Berger, Cathleen E Matrai, Tessa Cigler, Melissa K Frey

**Affiliations:** 1 Hadassah-Hebrew University School of Medicine, Faculty of Medicine, The Hebrew University of Jerusalem, Ein Kerem, PO Box 12271, Jerusalem, 9112102, Israel; 2 Weill Cornell Medicine, New York, NY 10065, USA

**Keywords:** breast neoplasms, endometrium, uterine bleeding, hysterectomy, anaemia, palliative care

## Abstract

Breast cancer is the most prevalent cancer in the United States. With an increasing rate of survivorship and extended life span for patients with metastatic disease, the demand for palliative care is increasing. Although uncommon, metastases to gynaecologic organs have been reported and are often present with post-menopausal bleeding. Post-menopausal bleeding can become clinically significant and have a detrimental effect on quality of life. We report the case of a 70-year-old woman with symptomatic vaginal bleeding caused by breast cancer metastatic to her uterus, cervix, fallopian tubes and ovaries. She was successfully treated with minimally invasive hysterectomy, resolving her vaginal bleeding and anemia and allowing her to resume chemotherapy.

## Introduction and discussion

Breast cancer is the most prevalent cancer in the USA with an estimated 252,710 new diagnoses in 2017 and 40,610 recorded deaths. Six per cent of patients have distant metastases at the time of diagnosis [1]. Breast cancer survivorship has been steadily increasing over the past several decades, owing to both earlier diagnoses and multiple treatment options, making breast cancer survival rates higher than most other cancer types [2]. The 5-year, 10-year and 15-year relative survival rates for breast cancer are 89%, 83% and 79%, respectively, leading to an estimated prevalence of more than 3.5 million women in USA with a history of breast cancer [3]. This improving survivorship results in an increased number of women living with metastatic breast cancer, with approximately 154,794 such women currently alive in the USA [4]. The increasing number of women living with metastatic disease requires continued attention and innovation in palliative care.

The most common sites of breast cancer metastases are the bone, lung, pleura, soft tissue and liver [5]. Metastases to the genital tract are rare; cancer that invades the uterus usually does so by direct extension and distant metastases are seldom found. However, when metastases to the uterus from remote origins are discovered, breast cancer is the most commonly discovered cancer (46.5% of cases) [6]. Although lobular invasive carcinoma is more likely to present with distant metastases to the female genital tract, invasive ductal carcinoma has been reported [7]. Breast cancer cells have been discovered in the myometrium and endometrium [6] as well as in endometrial polyps [8] and, rarely, even inside the leiomyosarcoma of the uterus [9, 10]. Although uncommon, metastases to the uterine corpus are an established pathological phenomenon. Uterine metastases are most likely to involve the myometrium and remain asymptomatic for a relatively long duration; however, when the endometrium is involved, vaginal bleeding can occur [11, 12, 13].

We report the case of a 70-year-old gravida seven para five female, with a history of metastatic lobular breast cancer, positive for estrogen receptor (ER) and progesterone receptor (PR) staining, and negative for human epidermal growth factor receptor 2 staining. Her breast cancer was initially diagnosed 5 years prior, and at the time of diagnosis involved the axilla and bones.

**Figure 1.**
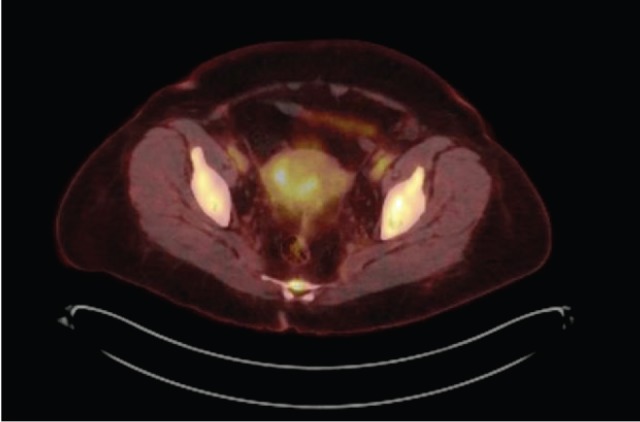
Increased FDG uptake on PET CT including uptake in uterus. PET CT scan. 1. Interval increase in FDG uptake of multiple hypermetabolic osseous metastases throughout the axial and proximal appendicular skeleton. 2. Mildly increased FDG uptake of mildly hypermetabolic mediastinal lymph nodes. 3. Moderate FDG uptake of the uterus centrally that was not present on imaging performed ten months prior.

**Figure 2.**
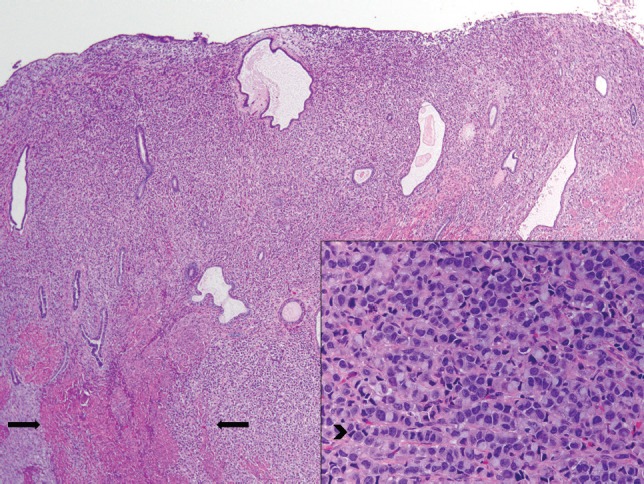
Breast metastasis in uterus corpus, endometrium and myometrium. Low-power image of uterus. There are sheets of neoplastic cells replacing the endometrial stroma and intersecting between muscle fibres of the myometrium (arrow). The tumour extended through the entire thickness of the myometrium to the uterine serosa. Cervical and adnexal involvement was also present. On high power (inset), the cells display areas of a classic invasive lobular carcinoma growth pattern, with single-filing of cells readily observed (arrowhead).

**Figure 3.**
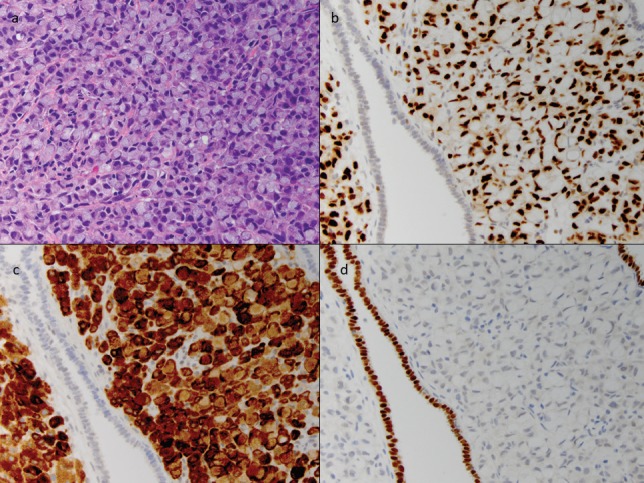
Cellular classification of tumour with immunohistochemistry. A high-power image of the tumour cells (a) shows abundant intracellular mucin displacing the nucleus to the periphery of the cell, imparting a 'signet ring' appearance. This feature was also noted in the prior axillary specimen. The tumour cells are strongly positive for GATA3 (b) and mammoglobin (c), two markers of breast origin, while the adjacent endometrium is negative. The cells do not stain for Pax-8 (d), a marker of Mullerian origin, while the intervening endometrium serves as a strong positive control. Additionally, approximately 1–10% of the tumour cells showed weak ER staining and less than 1% were positive for PR (not shown).

The patient received multiple treatments, including Letrozole, Zoledronic Acid, Exemestane, Fulvestrant and Vinorelbine. Approximately 5 years after her cancer diagnosis, while being treated with Capecitabine, she developed vaginal bleeding. She underwent a pelvic exam, which was notable for a small mobile uterus, without active vaginal bleeding or adnexal masses. PET CT scan demonstrated progression of the disease in her bone (axial and proximal appendicular skeleton) and mediastinal lymph nodes and moderate fluorodeoxyglucose (FDG) uptake of the uterus centrally that was not present on imaging performed ten months prior (Figure 1). The patient underwent uterine dilatation and curettage and hysteroscopy. The intra-operative findings were notable for a large friable polypoid mass filling the endometrial cavity and pathologic examination of the endometrial mass revealed metastatic breast cancer (Figures 2 and 3).

Following the procedure, the patient’s vaginal bleeding resolved, and she was started on Navelbine. As the patient’s bleeding had resolved and she required systemic chemotherapy, no further gynaecologic procedure was planned. However, 3 months later, she presented to the emergency room with heavy vaginal bleeding and symptomatic anemia (hemoglobin 6.7mg/dl and hematocrit 21.7g/dl). She received a transfusion of packed red blood cells, but continued to experience vaginal bleeding. After a thorough review of her clinical status, prognosis, and the management options, she elected to proceed with a palliative hysterectomy. The patient underwent robotic-assisted total laparoscopic hysterectomy and bilateral salpingo-oophorectomy. The patient tolerated the procedure well with minimal blood loss and was discharged home on the same day. The final pathologic examination demonstrated metastatic breast cancer present in the bilateral ovaries, fallopian tubes, uterus (endometrium, myometrium and serosa) and cervix. The patient recovered well from surgery and continued chemotherapy. Her hemoglobin and hematocrit at her post-operative visit were 9.2 mg/dl and 27.8 g/dl, respectively. At 8 months following the surgery, the patient had experienced no further episodes of vaginal bleeding or anemia.

## Conclusions

With the increasing prevalence of breast cancer, improvements in detection and the expanding spectrum of therapeutic options, a greater need has arisen for palliative care. Here, we present a case of rare metastases from breast cancer to the uterine corpus and endometrium that resulted in heavy vaginal bleeding and symptomatic anemia, and was successfully treated with minimally invasive hysterectomy. With the decreasing risk and morbidity of surgery owing to advancing surgical techniques, surgical palliation becomes an increasingly appealing option for some patients. We show that hysterectomy can provide long-term palliation for vaginal bleeding due to metastatic disease and should be considered for good surgical candidates when appropriate.
